# Bayesian spatio-temporal modeling of mortality in relation to malaria incidence in Western Kenya

**DOI:** 10.1371/journal.pone.0180516

**Published:** 2017-07-13

**Authors:** Sammy Khagayi, Nyaguara Amek, Godfrey Bigogo, Frank Odhiambo, Penelope Vounatsou

**Affiliations:** 1 Kenya Medical Research Institute-Center for Global Health Research, Kisumu, Kenya; 2 Swiss Tropical and Public Health Institute, Basel, Switzerland; 3 University of Basel, Basel, Switzerland; Centro de Pesquisas Rene Rachou, BRAZIL

## Abstract

**Introduction:**

The effect of malaria exposure on mortality using health facility incidence data as a measure of transmission has not been well investigated. Health and demographic surveillance systems (HDSS) routinely capture data on mortality, interventions and other household related indicators, offering a unique platform for estimating and monitoring the incidence-mortality relationship in space and time.

**Methods:**

Mortality data from the HDSS located in Western Kenya collected from 2007 to 2012 and linked to health facility incidence data were analysed using Bayesian spatio-temporal survival models to investigate the relation between mortality (all-cause/malaria-specific) and malaria incidence across all age groups. The analysis adjusted for insecticide-treated net (ITN) ownership, socio-economic status (SES), distance to health facilities and altitude. The estimates obtained were used to quantify excess mortality due to malaria exposure.

**Results:**

Our models identified a strong positive relationship between slide positivity rate (SPR) and all-cause mortality in young children 1–4 years (HR = 4.29; 95% CI: 2.78–13.29) and all ages combined (HR = 1.55; 1.04–2.80). SPR had a strong positive association with malaria-specific mortality in young children (HR = 9.48; 5.11–37.94), however, in older children (5–14 years), it was associated with a reduction in malaria specific mortality (HR = 0.02; 0.003–0.33).

**Conclusion:**

SPR as a measure of transmission captures well the association between malaria transmission intensity and all-cause/malaria mortality. This offers a quick and efficient way to monitor malaria burden. Excess mortality estimates indicate that small changes in malaria incidence substantially reduce overall and malaria specific mortality.

## Introduction

Morbidity and mortality estimates over the last decade across age groups in sub Saharan Africa (SSA) remain high compared to other regions despite an overall global reduction. The biggest burden is due to infectious diseases that largely affect children below 5 years of age with one of the main drivers of these consistently high estimates being malaria [1,2]. Recent studies and estimates show that malaria in SSA has reduced considerably, with a drop of over 37% for cases and 60% of deaths between the years 2000 and 2015 [2,3]. In western Kenya, the Kenya Medical Research Institute\Centers for Disease Control and Prevention’s Health and Demographic Surveillance System (KHDSS) has shown that between 2003 and 2010 there was a 67% reduction in malaria mortality in all ages and 70% in children below the age of 5 years even though it remains a leading cause of death [4].

Malaria infection is driven by different factors and measuring its burden has largely been problematic, especially in areas where the disease’s health impact is huge. Malaria transmission intensity is an important measure of this burden and can largely be classified into infection in humans (parasite prevalence and incidence rates), interaction between mosquitos and humans (entomological inoculation rate (EIR)) and vector measures like (mosquito density, vectoral capacity and sporozoite rate). The links between the above drivers of transmission and mortality still need further investigation so as to understand and quantify them well. Recent studies have concentrated on measures of vector density and interaction between mosquitos and humans to measure transmission intensity and link it to both all-cause and malaria specific mortality [5–8]. Meanwhile measures of infection in humans have largely been carried out either through parasite prevalence or disease rates as these can be done as one-off surveys with ease especially during peak transmission times. Incidence as a measure of transmission has largely been unused since it requires more investment of time and resources. Ensuring the correct denominator is used while taking into account health seeking behavior, poor health systems and diagnostic challenges limit its usability and makes quality data unavailable.

Slide positivity rate (SPR), has been used as a surrogate for malaria incidence and a key indicator of temporal trends in malaria disease burden. It measures the proportion of slide confirmed malaria positive cases out of cases examined, and has been shown to be a good predictor of incidence [9–11]. It is relatively inexpensive compared to other measures of transmission and easy to monitor at sentinel health facilities hence a useful measure of not only trends but the overall burden of malaria disease.

Despite global improvements in data collection and use, there is a large gap of adequate and quality data on malaria cases and deaths in low and middle income countries. Estimates provided from these data are fraught with inherent differences in data collection methodology, analysis and interpretations This has been attributed to poor health surveillance systems that are incapable of collecting quality data to be used in informing policy [12,13]. It is important therefore to explore and determine the best modes of appropriately measuring malaria burden so as to allow for accurate determination of progress and assess the contribution of different interventions especially at local levels. With this in mind, health and demographic surveillance system (HDSS) sites were established in different regions of SSA, Asia and Oceania to supplement efforts in providing accurate data on demography and public health [14,15]. These HDSS sites have accumulated a wealth of information that is well aligned in both space and time, offering a unique platform to monitor and provide accurate estimates of disease at a local level. The other benefit that HDSS sites confers is the intensity and longevity of its operations which provide consistently regular longitudinal data that can aid policy making in resource strained settings[16]. In addition to household survey data, HDSS sites, using verbal autopsy (VA) collect data on morality that is used to determine cause of death, which is an important tool, especially where conventional autopsy data is not available [17]. HDSS sites are therefore an important platform for malaria burden estimation.

The Malaria Transmission Intensity and Mortality Burden across Africa (MTIMBA) project sought to analyze data on malaria transmission intensity and burden in Africa from 2002 to 2004. Project results indicated that malaria transmission as measured by EIR drives mortality especially in younger children and showed the importance of small scale heterogeneity in estimating malaria related deaths. However their analysis did not include data on malaria morbidity or interventions. Furthermore, the two year duration of the project could not allow adequate assessment of trends and changes in malaria mortality in relation to interventions.

In this study, malaria indicator incidence data collected from selected health facilities was linked to household data so as to improve estimates of malaria related burden by including more malaria transmission indicators over a period of 6 years in the Kenya Medical Research Institute\Centers for Disease Control and Prevention’s Health and Demographic Surveillance System (KHDSS) of western Kenya. We also sought to explore the relationship between slide positivity rate from these health facilities as a measure of incidence and all-cause/malaria-specific mortality derived from verbal autopsy (VA). Bayesian geostatistical spatio-temporal models were used to estimate the contribution of SPR to malaria mortality in an area of almost year round transmission.

## Materials and methods

### Study area and population

**Fig 1:**
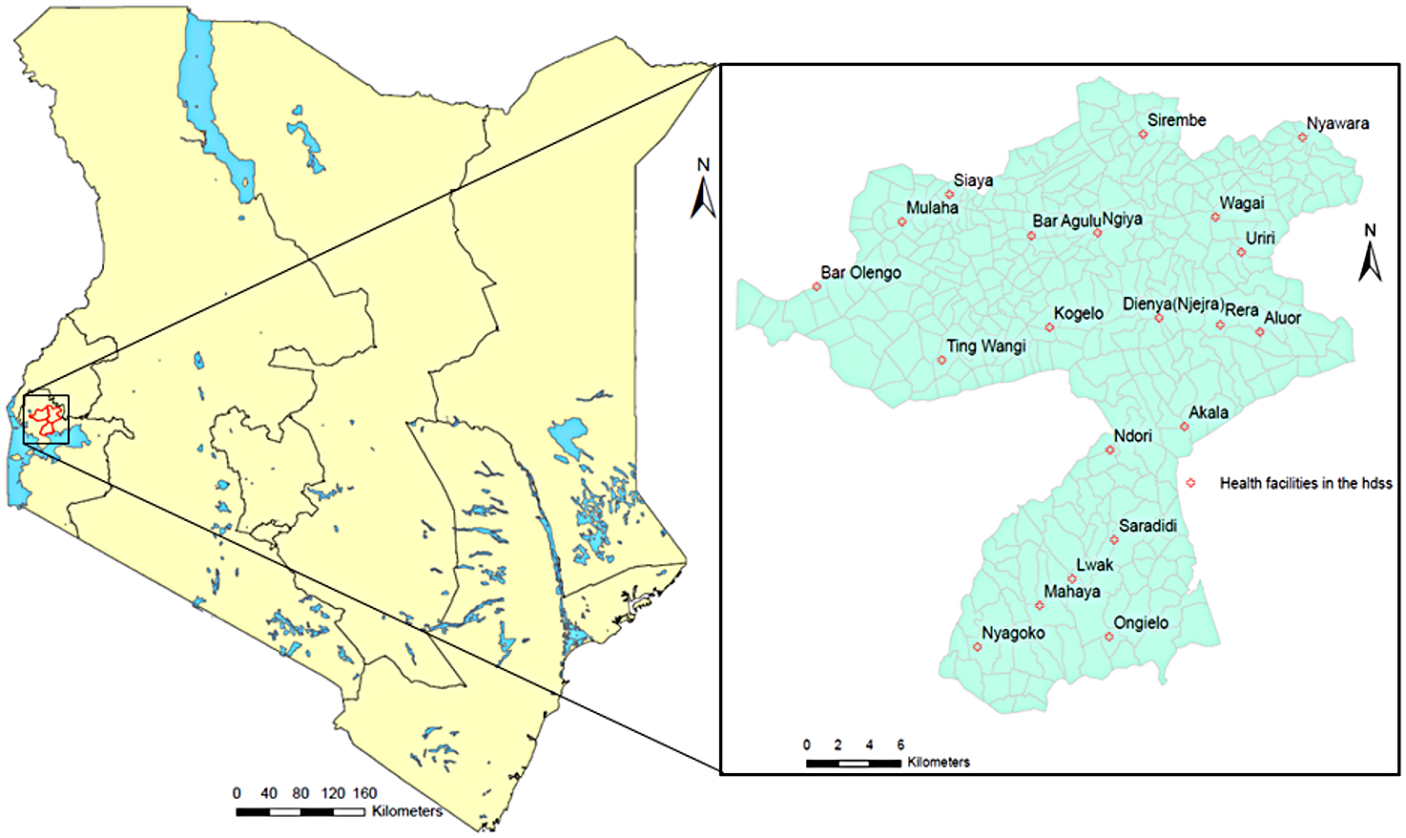
KHDSS study area in western Kenya showing villages and sentinel health facilities.

The KHDSS has been described in details elsewhere [14,18]. In brief, the KHDSS is located in three regions (Asembo, Gem and Karemo) of rural western Kenya, Siaya County (Fig 1). The study area borders the northern shores of Lake Victoria; is spread over 700km^2^ divided into 385 villages with a mid-year population of over 240,600 people in 58,700 households. The population is predominantly one ethnic group who derive a big part of their livelihood from subsistence farming and fishing. It is located in a malaria endemic zone having round the year transmission with peaks in May-June and November-December [5]. Infectious diseases and HIV/AIDS are the other important causes of morbidity and mortality [14,18,19].

### Slide positivity rates

Children aged below 14 years admitted at selected sentinel health facilities within the KHDSS (Fig 1) from January 2007 to December 2012 with history of or documented fever were screened for malaria infection by collecting a blood slide for microscopy examination regardless of their residency status. Blood smears were collected and screened microscopically after staining with giemsa. Presence of parasites and reported or documented fever was classified as malaria infection. All the microscopists at these health facilities are evaluated after every 3 months for competency in reading malaria microscopy slides and 10% of the slides are rechecked for quality control. However for this analysis, we restricted our numbers to patients registered and residing in the study area to calculate the slide positivity rate (SPR). The data were aggregated at monthly intervals and the slide positivity rate (SPR) calculated as the proportion of malaria positive cases as determined by microscopy out of the total cases tested.

### KHDSS surveillance data and verbal autopsy

The KHDSS population has been monitored from an initial census in 2001at household level to establish the number of people in a geographically defined region followed by tri-annual visits after every 4 months to establish any changes due to migration, deaths or births. In addition, other data was collected at household level for socio-economic indicators, house types and insecticide-treated net (ITN) ownership.

The verbal autopsy (VA) methodology and data used in this study has been described in details elsewhere [20,21]. After death of a registered KHDSS resident, a notification is filled as soon as possible by a reporter based in the same village. At least 3 weeks are given to allow for mourning before an interview is conducted. A detailed questionnaire is printed to collect data on the deceased last disease, signs, symptoms and medical history. A trained interviewer looks for the most appropriate interviewee who was closest to the deceased and knew about the illness, disease or condition that led to death to administer the questionnaire. The data is then captured to an electronic database, validated and processed using the InterVA program which is a computer based expert opinion algorithm that is based on the Bayes theorem. It uses probabilistic methods to interpret verbal autopsy data using *a priori* approximations of probabilities related to diseases and last symptoms exhibited by the diseased so as to determine the most probable cause of death [21] based on these data. Output from the InterVA model was used to determine the most probable causes of death including malaria.

Data on household assets ownership and house characteristics was used to create a composite socio-economic status index using the multiple correspondence analysis (MCA) technique as described in previous studies [22]. The household scores were then aggregated at village level and ranked into 3 categories, i.e. least poor for the well off, poor for the average and poorest for the lowest rank. Bed net ownership was used as a measure of existing interventions that have been implemented in the area. This was calculated as the percentage of households per village owning at least one net for every two people as recommended by the WHO [23]. The data used are described in details in S1 File

### Statistical analysis

All registered residents in the KHDSS villages during the period 2007 to 2012 were included in the analysis. To qualify as a resident, a person has to stay continuously in the study area for at least 3 calendar months or is born to a resident mother while she is a resident. The participants were grouped into 6 age groups as follows: 0–28 days (neonates), 1–11 months (infants), 1–4 years (child), 5–14 years (older child), 15–59 (adults) and 60+ (elderly).

Person time at risk in months was calculated as the total time spent in the study area from date of enrollment until they exited through outmigration or death; alternatively they stopped being observed due to loss to follow-up or reached the end of the observation period set at 31^st^ December 2012.

Crude and age specific all-cause mortality and malaria rates were calculated by dividing the deaths in each group with the total person years observed (pyo). For each age group, Cox proportional hazards models were approximated using negative binomial regression [24] with time to death in person months as discrete contribution of each resident summed up at village level. Initial exploratory analysis was carried out in Stata 14 (Stata Corporation USA) to assess bivariate models of the relationship between the different factors and all-cause or malaria specific mortality. We included all the variables that were at 20% statistical significance in bivariate analysis into the Bayesian spatiotemporal negative binomial regression model. Variables included in the model were incidence risk by age group and village, age, bed net ownership, distance to health facilities, altitude, socio-economic status, area of residence and year of study. Spatial variation was included in the model as village specific random effects through latent observations of a spatial Gaussian process with mean zero and a covariance that assumed an exponential variation function of distance between two villages [25]. Temporal variation was modeled by a first order autoregressive process using monthly random effects. Bayesian models were fitted in OpenBugs version 3.1.2 (Imperial College and Medical Research Council London, UK) using Markov Chain Monte Carlo (MCMC) simulation. Covariate effects from the Bayesian geo-statistical model were considered statistically important when the credible intervals (CI) of the estimated regression coefficients did not include zero. Due to the nature of Bayesian statistical inference, we replace the terminology statistically significant by statistically important when reporting our results. Details of the Bayesian geostatistical temporal model are given in the supplement file S2 File—Bayesian model formulation.

### Excess mortality attributed to slide positivity

To quantify the excess mortality rate (EMR), we used model coefficients for each age category to determine the mortality rates at different levels due to slide positivity rate (SPR). We calculated the difference between the mortality rates (MR) when SPR is greater than zero and when SPR is equal to zero.

EMR=MR(SPR>0−MR(SPR=0)

The calculated EMR values were then plotted against SPR between 0.001 and 100 at intervals of 0.005.

The study protocol for the KHDSS has been approved by the Kenya Medical Research Institute (#1801) and Centers for Disease Control and Prevention (#3308) institutional review boards. Written informed consent was obtained from the heads of compounds in conducting the household interviews while hospital data was only collected from individuals who also gave an additional written informed consent or from the caretaker in-charge if the participants were under 18 years of age. Data were obtained with permission of the KEMRI/CDC HDSS Steering committee. The data are available from the Kenya Medical Research Institutes' Institutional Data Access / Ethics Committee for researchers who meet the criteria for access to confidential data. Data are from the HDSS study whose authors may be contacted at Gbigogo@kemricdc.org or Skhagayi@kemricdc.org. This article is published with the permission of the director Kenya Medical Research Institute. The KHDSS is a member of the INDEPTH Network.

## Results

### Descriptive statistics

During the period January 2007 to December 2012, there were a total of 375,447 uniquely registered residents in the study area contributing a total of 1,360,933 person years of observation (pyo). We observed an overall crude death rate of 13.8 deaths per 1,000 pyo, with a high of 18.8 deaths per 1,000 pyo in 2008 to a low of 10.2 deaths per 1,000 pyo in 2012. Malaria mortality was at 2.1 deaths per 1,000 pyo over the same period and followed a similar trend to the all-cause mortality rates. Alternatively slide positivity rate (SPR) during the same period was 52.5% and followed a similar trend to the mortality rates over the years as shown in Table 1.

**Table 1:** All-cause mortality, malaria specific mortality and malaria risk rates by year.

Year	Slide positivity rate (%)	Person years of observation*	All-cause death rate per 1,000 pyo (95% CI)	Malaria death rate per 1,000 pyo (95% CI)
2007	41.3%	181537	15.5 (14.9, 16.1)	1.3 (1.2, 1.5)
2008	58.0%	230374	18.8 (18.2, 19.3)	3.5 (3.3, 3.7)
2009	59.0%	230373	15.6 (15.1, 16.2)	2.9 (2.6, 3.1)
2010	56.3%	233871	12.4 (11.9, 12.8)	2.1 (1.9, 2.3)
2011	47.5%	238524	10.9 (10.5, 11.3)	1.4 (1.2, 1.5)
2012	46.4%	246254	10.2 (9.8, 10.6)	1.4 (1.2, 1.5)
**Overall**	**52.5%**	**1360933**	**13.8 (13.6, 14.0)**	**2.1 (2.0, 1.3)**

*person years of observation (pyo) used for calculating all cause and malaria specific mortality only

**Fig 2:**
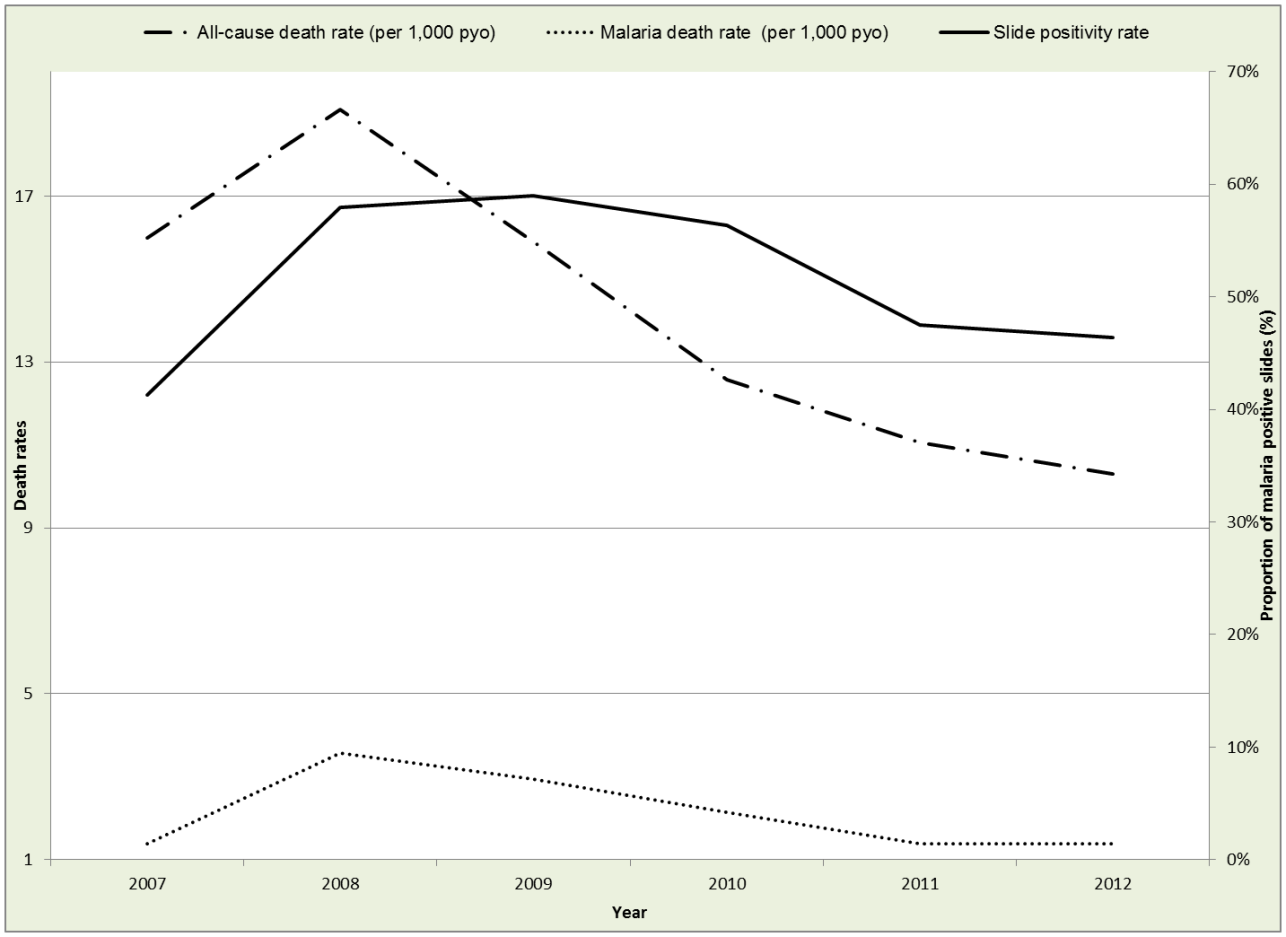
Yearly all-cause and malaria specific death rates versus malaria risk.

**Fig 3:**
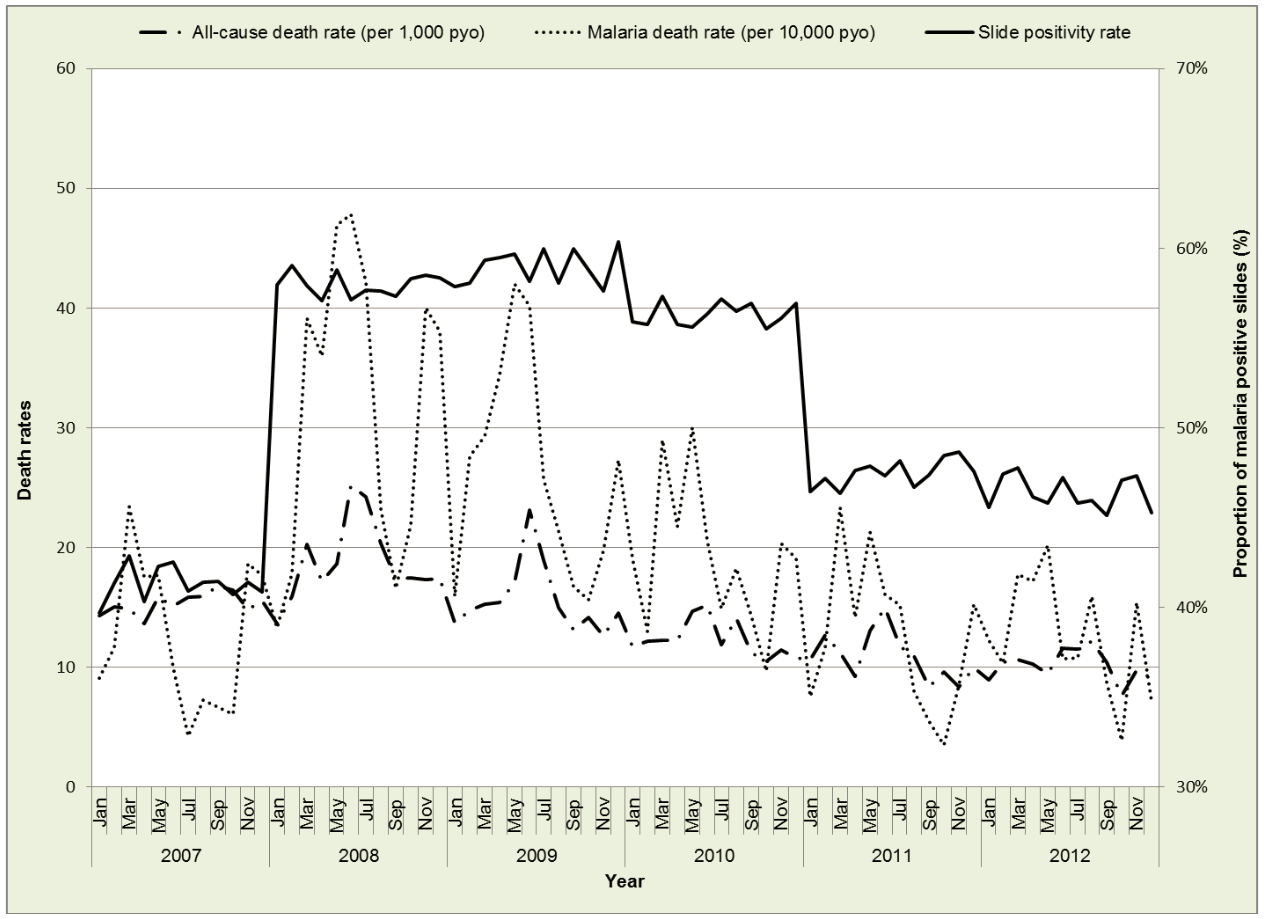
Monthly all-cause/malaria specific mortality versus slide positivity rate.

Figs 2 and 3 show the yearly and monthly trends in all-cause deaths, malaria specific deaths and malaria risk. The monthly trends show some peaks for all-cause and malaria specific deaths but not so distinct for SPR. The SPR rose sharply between December 2007 and January 2008 and stayed at these levels until the end of 2010, when there was a gradual drop from 2011 to 2012.

**Fig 4:**
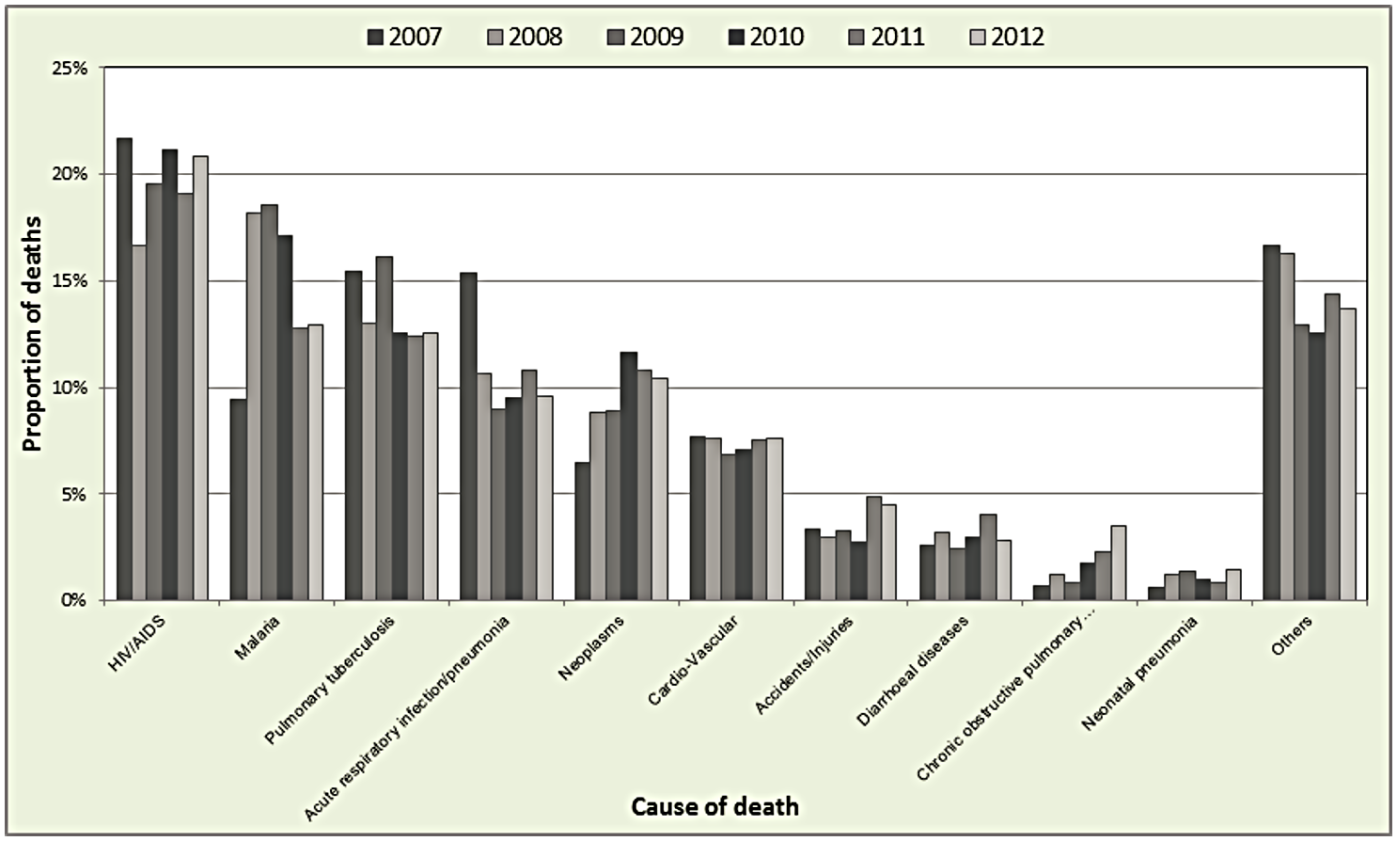
Main causes of death in the KHDSS.

Out of the total 18,729 deaths, 17,016 (91%) had cause of death assigned by the InterVA. The top causes of deaths in the whole population were HIV/AIDS, malaria and pulmonary tuberculosis (Fig 4). Over the years, there was a downwards trend for most of the diseases as a proportion of overall deaths, there was however an upwards spike in malaria deaths in the years 2008/2009 which coincided with a drop for HIV/AIDS, acute respiratory infections, cardio-vascular illnesses and those grouped as others.

**Fig 5:**
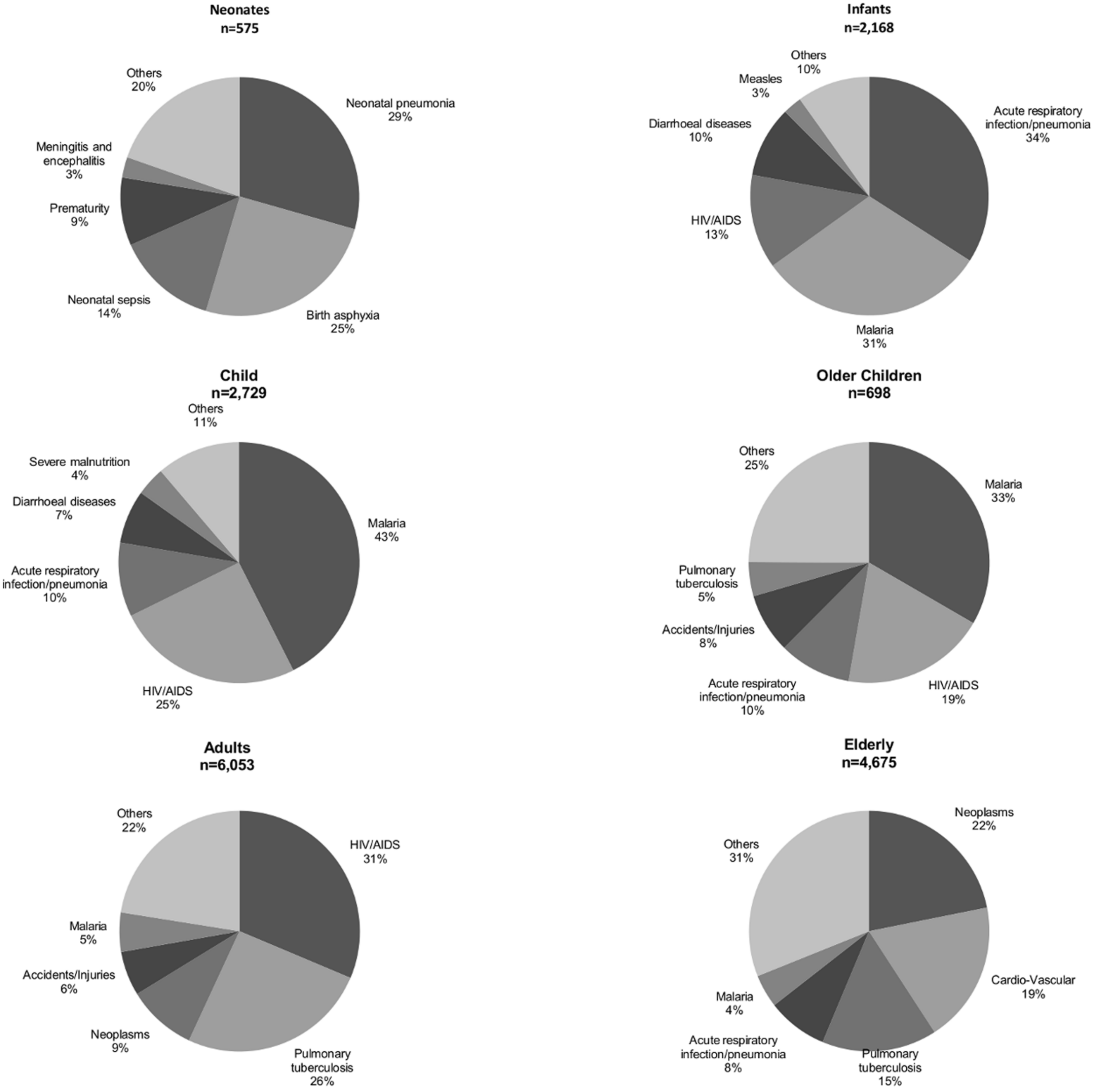
Main causes of death in the KHDSS by age groups.

Fig 5 depicts the distribution of main causes of deaths including malaria among the different age groups. The top causes of mortality were pneumonia and births asphyxia for neonates, acute respiratory infection/pneumonia and malaria for infants, malaria and HIV/AIDS for both younger and older children, HIV/AIDS and pulmonary tuberculosis for adults, while for the elderly, the main causes were neoplasms and cardio-vascular diseases. Overall, the proportion of malaria was highest in the child age group (45%), followed by older children (33%) and infants (31%).

ITN ownership, distance to health facilities, socio-economic status, year of study, region and altitude were associated with either all-cause or malaria specific mortality in at least one of the age groups. For comparability purposes, these were included in all the seven Bayesian geostatistical models in both malaria-specific and all-cause mortality analysis.

### Model based results

Table 2 shows the results from the Bayesian spatio-temporal negative binomial regression models for all-cause mortality by age groups at village levels. SPR was positively associated with all-cause mortality among infants, child, elderly and combined age group analysis. However, this association was important in the child (HR = 4.29; 95% CI: 2.78–13.29) and the combined age-group analysis (HR = 1.55; 95% CI: 1.04–2.80). SPR was not associated with all-cause mortality in neonates, older children and adults.

**Table 2:** Posterior estimates (median) of the hazard ratio (HR) for predictors of all-cause mortality by age categories.

Covariates	Neonates	Infants	Child	Older child	Adults	Elderly	Overall**
	(0-28days)	(1-11month)	(1-4yrs)	(5-14yrs)	(15-59yrs)	(60 +)	
	HR	HR	HR	HR	HR	HR	HR
	(95% CI*)	(95% CI)	(95% CI)	(95% CI)	(95% CI)	(95% CI)	(95% CI)
Malaria risk	0.89	3.10	4.29	0.48	0.73	1.70	1.55
	(0.13–5.70)	(0.36–13.12)	(2.78–13.29)	(0.15–2.05)	(0.39–1.42)	(0.79–4.45)	(1.04–2.80)
ITN	1.01	1.00	1.00	0.99	1.00	0.99	1.00
	(1.00–1.02)	(0.99–1.01)	(0.99–1.003)	(0.98–1.01)	(0.997–1.002)	(0.99–1.00)	(0.998–1.001)
Distance to health facility							
0–1 km	1	1	1	1	1	1	1
1–2 km	1.21	1.24	1.12	1.01	1.15	1.00	1.11
	(0.91–1.63)	(1.09–1.42)	(1.01–1.26)	(0.81–1.27)	(1.06–1.25)	(0.90–1.11)	(1.05–1.17)
>2 km	1.49	1.23	1.27	1.13	1.09	1.03	1.12
	(1.11–2.11)	(1.07–1.43)	(1.12–1.44)	(0.93–1.45)	(0.99–1.20)	(0.92–1.16)	(1.05–1.20)
Ses							
Poorest	1	1	1	1	1	1	1
Poor	0.91	1.08	0.92	0.81	0.94	1.03	0.98
	(0.70–1.17)	(0.95–1.23)	(0.83–1.03)	(0.64–1.01)	(0.86–1.02)	(0.94–1.13)	(0.93–1.03)
Least poor	0.64	0.97	0.79	0.83	0.94	0.99	0.94
	(0.48–0.84)	(0.84–1.11)	(0.70–0.88)	(0.66–1.03)	(0.86–1.05)	(0.90–1.09)	(0.89–0.99)
Year							
2007	1	1	1	1	1	1	1
2008	1.25	1.29	1.23	1.31	1.05	1.07	1.15
	(0.77–2.14)	(0.39–2.24)	(1.02–1.70)	(0.93–2.18)	(0.75–1.55)	(0.77–1.47)	(0.82–1.55)
2009	0.84	1.07	0.92	1.24	0.91	1.00	1.04
	(0.49–1.47)	(0.28–1.85)	(0.69–1.19)	(0.73–2.13)	(0.64–1.33)	(0.72–1.44)	(0.67–1.48)
2010	0.54	0.84	0.74	1.00	0.73	0.92	0.88
	(0.30–0.98)	(0.21–1.53)	(0.52–0.97)	(0.57–1.73)	(0.50–1.09)	(0.68–1.38)	(0.58–1.66)
2011	0.55	0.81	0.68	0.68	0.59	0.96	0.84
	(0.31–0.97)	(0.25–1.81)	(0.47–0.92)	(0.38–1.12)	(0.41–0.89)	(0.70–1.44)	(0.57–2.01)
2012	1.02	0.72	0.62	0.85	0.56	0.89	0.80
	(0.61–1.72)	(0.27–1.85)	(0.43–0.87)	(0.47–1.46)	(0.38–0.83)	(0.61–1.24)	(0.49–1.84)
Altitude							
1147–1243	1	1	1	1	1	1	1
1244–1293	1.41	1.07	1.07	0.95	0.97	0.96	0.97
	(0.98–2.06)	(0.88–1.30)	(0.93–1.26)	(0.72–1.24)	(0.85–1.10)	(0.82–1.14)	(0.87–1.08)
1294–1327	1.33	0.85	0.94	0.74	0.84	0.91	0.85
	(0.90–2.00)	(0.68–1.08)	(0.80–1.14)	(0.54–1.02)	(0.72–0.99)	(0.74–1.13)	(0.75–0.97)
1328–1365	1.46	0.79	0.99	0.68	0.92	1.01	0.88
	(0.96–2.30)	(0.61–1.02)	(0.91–1.23)	(0.46–0.98)	(0.77–1.09)	(0.80–1.27)	(0.77–1.03)
>1365	1.43	0.75	0.89	0.65	0.88	1.01	0.87
	(0.91–2.35)	(0.55–1.01)	(0.75–1.14)	(0.42–0.94)	(0.72–1.07)	(0.78–1.31)	(0.74–1.03)
Spatial Variation	0.44	2.36	2.04	0.74	0.99	0.52	1.69
	(0.14–1.90)	(0.70–8.93)	(0.72–7.61)	(0.22–3.59)	(0.33–4.42)	(0.20–1.88)	(0.76–6.15)
Temporal Variation	0.14	0.10	0.11	0.13	0.06	0.07	0.05
	(0.08–0.25)	(0.07–0.17)	(0.07–0.17)	(0.08–0.22)	(0.04–0.08)	(0.04–0.10)	(0.04–0.08)
Range***	34.41	11.15	11.38	17.19	13.18	20.79	11.40
	(8.77–95.37)	(8.10–39.18)	(8.11–40.52)	(8.27–84.45)	(8.16–55.67)	(8.45–83.93)	(8.12–40.42)

* CI = credible interval

** In addition to the variables above we also adjusted for age in the overall model

*** Minimum distance in kilometers at which spatial variation is statistically important at 5%

Average distance to health facilities was positively associated with increased all-cause mortality among all age groups. We observed higher mortality rates for distances greater than 1km or greater than 2km on average from health facilities. The association was important for neonates, infants, child and the combined analysis. Among the 3 levels of socio-economic status, there was reduced mortality risk in all age group with higher socio-economic status. The highest socio-economic status was associated with reduced all-cause mortality in neonates, children below 5 years and in the combined age group analysis. We observed a higher risk of mortality in the year 2008 compared to 2007 for all the ages which then reduced in the subsequent years and followed a downwards trend; however, in 2012 there was a slight reversal back to the pre-2008 levels among the neonates even though not statistically important. Higher altitude had a negative association with mortality in all the groups except the elderly and neonates but this association was only statistically important in the older children, adults and the combined age groups.

Estimated spatial variation at village level was higher than temporal variation in all age groups except the elderly. The minimum distance at which spatial variation was statistically important at 5% ranged from 11.5 to 34.42.

In modelling malaria-specific mortality in relation to SPR, we developed a Bayesian spatio-temporal model with similar variables used in the all-cause mortality model as shown in Table 3. Neonates were excluded from the malaria-specific modeling because there were no deaths attributed to malaria by InterVA in this group. Malaria mortality increased with an increase in SPR among infants, child and overall analysis. SPR was strongly associated with increased risk of malaria mortality among the child group (HR = 9.48; 96% CI: 5.11–37.94); however, in, it was strongly associated with a reduction in malaria specific mortality (HR = 0.02; 95% CI: 0.003–0.33).

**Table 3:** Posterior estimates (median) of the hazard ratio (HR) for predictors of cause-specific mortality by age categories.

Covariate	Infants	Child	Older child	Adults	Elderly	Overall**
	(1-11month)	(1-4yrs)	(5-14yrs)	(15-59yrs)	(60 +)	
	HR	HR	HR	HR	HR	HR
	(95% CI*)	(95% CI)	(95% CI)	(95% CI)	(95% CI)	(95% CI)
Malaria risk	1.36	9.48	0.02	0.27	0.59	1.37
	(0.23–9.85)	(5.11–37.94)	(0.003–0.33)	(0.02–3.24)	(0.01–13.15)	(0.51–3.73)
ITN	1.01	1.0	1.01	0.99	1.02	1.00
	(1.00–1.02)	(0.99–1.01)	(0.99–1.03)	(0.98–1.01)	(1.00–1.04)	(0.99–1.01)
Distance to health facility						
0–1 km	1	1	1	1	1	1
1–2 km	1.19	1.26	1.10	1.02	0.98	1.12
	(0.89–1.60)	(1.05–1.45)	(0.76–2.04)	(0.72–1.45)	(0.50–2.01)	(0.95–1.30)
>2 km	1.18	1.42	0.93	0.94	1.24	1.14
	(0.85–1.64)	(1.35–1.66)	(0.75–1.20)	(0.67–1.10)	(0.63–2.51)	(0.95–1.37)
Ses						
Poorest	1	1	1	1	1	1
Poor	1.03	0.94	1.01	1.03	1.10	0.99
	(0.79–1.37)	(0.77–1.08)	(0.55–1.46)	(0.66–1.58)	(0.60–2.13)	(0.86–1.15)
Least poor	0.94	0.76	0.58	1.27	0.87	0.92
	(0.70–1.26)	(0.62–0.89)	(0.41–1.07)	(0.82–1.95)	(0.53–1.86)	(0.79–1.07)
Year						
2007	1	1	1	1	1	1
2008	9.32	2.34	1.26	1.60	1.73	2.12
	(3.34–57.79)	(1.36–4.07)	(0.63–4.03)	(0.79–3.36)	(0.49–2.67)	(0.93–4.14)
2009	7.21	1.57	4.59	2.13	3.28	1.86
	(2.56–47.57)	(1.08–2.51)	(1.81–7.23)	(1.52–4.36)	(1.07–58.62)	(0.57–3.89)
2010	6.95	1.87	5.19	1.43	5.75	2.02
	(2.36–45.32)	(1.18–2.70)	(1.85–15.48)	(0.70–2.54)	(1.66–15.5)	(0.55–4.02)
2011	6.07	1.74	0.77	0.79	3.00	1.45
	(1.99–37.2)	(1.25–2.87)	(0.46–2.48)	(0.32–1.71)	(1.16–30.4)	(0.32–3.02)
2012	5.31	2.09	2.09	0.95	2.68	1.60
	(1.66–36.72)	(1.33–3.19)	(1.01–6.14)	(0.43–2.05)	(0.99–46.27)	(0.34–3.28)
Altitude						
1147–1243	1	1	1	1	1	1
1244–1293	0.82	0.69	0.73	0.61	1.46	0.70
	(0.54–1.24)	(0.56–0.92)	(0.39–1.62)	(0.28–1.14)	(0.93–5.25)	(0.54–0.93)
1294–1327	0.63	0.72	1.14	0.71	1.04	0.68
	(0.40–1.03)	(0.57–0.98)	(0.55–2.43)	(0.39–1.31)	(0.28–3.73)	(0.50–0.92)
1328–1365	0.58	0.71	0.65	0.73	1.63	0.64
	(0.35–0.99)	(0.56–1.01)	(0.42–1.64)	(0.37–1.42)	(0.55–5.65)	(0.45–0.88)
>1365	0.39	0.64	1.08	0.57	2.74	0.58
	(0.22–0.76)	(0.46–0.91)	(0.50–2.67)	(0.27–1.15)	(1.14–10.81)	(0.40–0.85)
Spatial Variation	5.87	5.66	0.73	0.46	0.40	8.83
	(1.00–29.11)	(1.30–21.34)	(0.22–5.14)	(0.17–2.91)	(0.15–1.81)	(4.11–29.28)
Temporal Variation	0.15	0.18	0.41	0.29	0.42	0.17
	(0.09–0.28)	(0.11–1.30)	(0.16–1.28)	(0.14–0.61)	(0.17–1.38)	(0.11–0.27)
Range***	10.95	10.74	18.37	30.11	36.0	10.9
	(8.09–40.52)	(8.08–36.21)	(8.27–82.37)	(8.56–93.64)	(8.83–95.26)	(8.09–34.21)

* CI = credible interval

** In addition to the variables above we also adjusted for age in the overall model

*** Minimum distance in kilometers at which spatial variation is statistically important at 5%

Similar to the all-cause mortality above, reduced average distance to health facilities, higher socio-economic status and year of study were associated with reduced risk of malaria mortality.

In all models, spatial variation was higher than temporal variation with the exception of the model corresponding to the 60+ age group. The minimum distance at which spatial variation for malaria mortality by village was statistically important at 5%, ranged from 10.74km in the child age group to a high of 36km among the elderly.

**Fig 6:**
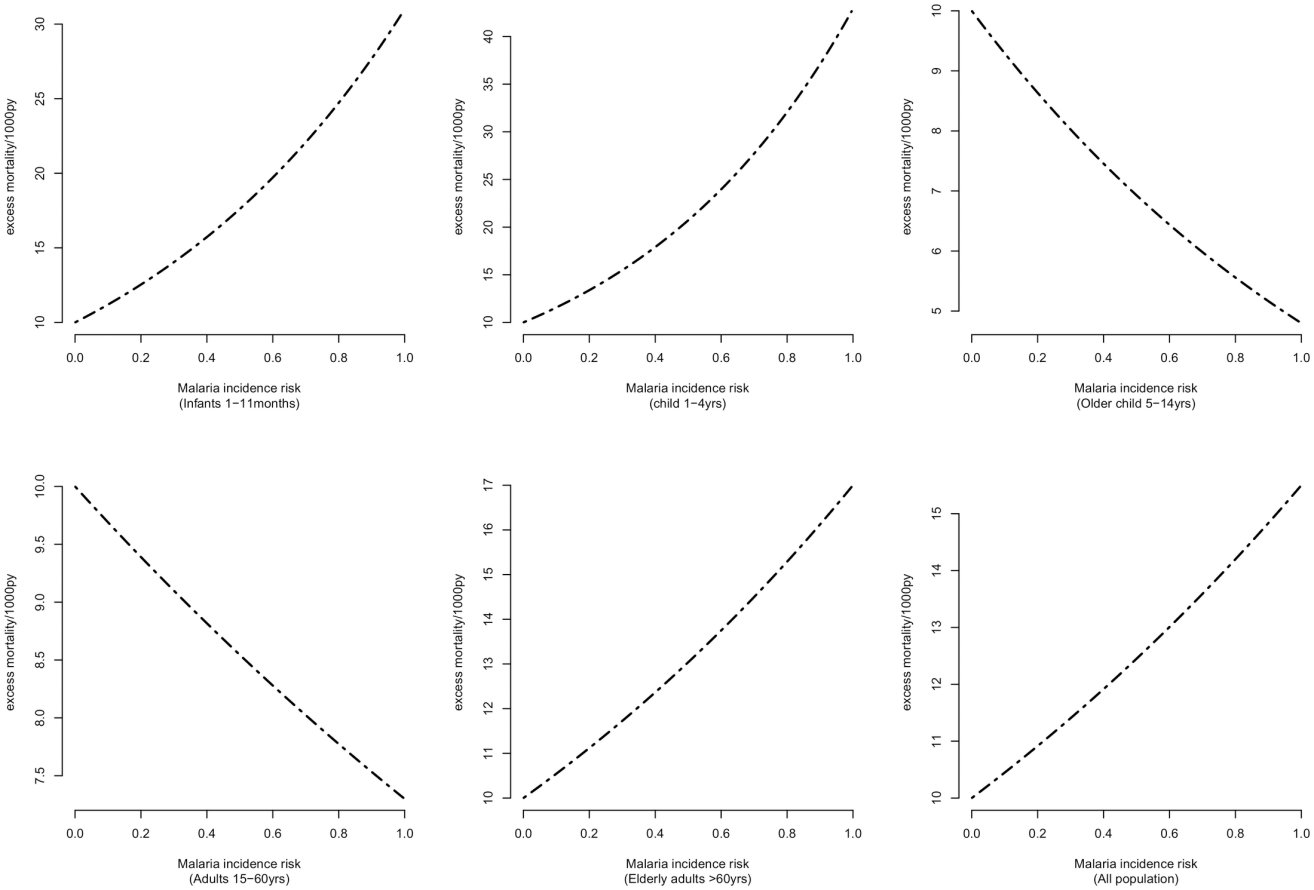
Age specific patterns of excess mortality due to malaria incidence.

Fig 6 shows the excess mortality as a function of malaria transmission. In infants, children 1–4 years, elderly adults and the combined population we noted an increase in mortality rate with SPR. However a protective effect was noted among children 4–14 years and adults 15–59 years. The highest burden was noted among children 1–4 years and infants 1–11 months.

## Discussion

Several studies have investigated the effect of malaria transmission on mortality using mostly entomologic inoculation rate or prevalence data as a measure of transmission [5,6,8]. Entomological data is quite sparse while prevalence data does not reflect seasonal variations of transmission. Our study is the first to link HDSS malaria mortality and health facility incidence data collected continuously in a well-defined geographical area. Using these datasets well aligned in space and time, we investigated SPR as a measure of transmission in relation to all-cause and malaria-specific mortality in the KHDSS area of western Kenya employing rigorous Bayesian spatio-temporal models to account for variation in space and time. We adjusted for person time observed as discrete monthly intervals, socio-economic status, ITN ownership, average distance to health facilities, altitude and year of study. In estimating excess mortality due to malaria transmission, it was noted that small changes in slide positivity rate (SPR), results in significant increases in overall mortality in this population.

In modelling the relationship between SPR and mortality, we found that SPR predicts all-cause mortality in the whole population. Among different age-groups, SPR was an important measure of both all-cause and malaria-specific mortality for children aged 1–4 years implying that malaria infection in under 5 children contributes greatly to overall mortality. Other studies have also shown a positive association between malaria transmission and all-cause mortality in this age group [5,6,8,26], however, were focused mostly on the relationship between all-cause mortality and EIR or prevalence. Children aged 1–4 years are most affected by malaria mortality due to lower immunity compared to neonates or children below 6 [27]. The effect of SPR on malaria-mortality among children aged 1–4 years could also be a result of delayed access to treatment and the magnitude of malaria burden in this group. Decreasing malaria incidence through proper and timely treatment and management can therefore greatly reduce both all-cause and malaria specific mortality. Slide positivity rate as a measure of malaria exposure appears to be a protective factor for malaria-specific mortality in older children (5–14 years), most likely due to acquired immunity at lower ages [28,29]. This explanation is supported by data from the same area indicating that *Plasmodium falciparum* parasite prevalence is highest in this group (KEMRI/CDC unpublished data). These school going children are less mobile compared to younger children who move with their working parents [14], and therefore the constant exposure to infection increases their immunity. It is important to note that even though there was low overall mortality in older children; malaria deaths as a proportion of all deaths in this group were still high and increased over the years. Desai et al also reported that malaria deaths in the 5–14 age group increased over the years; an indication reflecting either behavioral factors or reduced attention towards this age-group from control programs that target mainly pregnant women and children under five years [4].

Our study supports earlier results by Amek at al. [5] in the same study area where EIR as a measure of transmission was seen to drive mortality in children under 5 years but not in the 5–14 age group. Of importance is that in our study, SPR (OR = 4.29 and OR = 9.48) shows a stronger effect than EIR (OR = 1.58 and OR = 1.97) for both all cause and malaria specific mortality respectively. With proper diagnosis and data collection, SPR can be collected more easily using fewer resources at local levels than EIR. This highlights the importance of incidence as a driver of malaria transmission and consequently a better measure of exposure that can be used to monitor progress towards malaria control in areas of high endemicity.

During the study period, there was an upward spike in all-cause mortality, malaria specific mortality and SPR in 2008, followed by a steady decline between 2009 and 2012. The mortality increase in the year 2008 was observed in previous studies carried out in the same area and attributed to the effect of post-election violence in Kenya at the end of 2007 [4,30]. The rise in all-cause/malaria-specific mortality and SPR could be attributed to these clashes, which disrupted health services, interfered with the supply and provision of antimalarial drugs and led to a surge in malaria among other infectious diseases. Our findings echo what has been observed in other conflict areas in Africa and demonstrated the impact of conflicts on malaria burden [31]. Malaria as a proportion of all deaths was most affected since its increase in 2008–2009 coincided with a drop in HIV/AIDS, acute respiratory infections and cardio-vascular diseases among others showing that malaria can easily bounce back more forcefully whenever control efforts are interrupted. Malaria-specific mortality reduction in the other age groups can be attributed to the scale up of interventions, increased coverage of ITNs, prompt and improved malaria treatment using artemisinin-based combination therapies (ACT) and indoor residual spraying [3,19,30]. All-cause mortality reduction could be attributed to a reduction in malaria-specific mortality, scaled up provision of antiretroviral therapy (ART) and improvements in health service delivery that saw declines in infectious diseases and HIV/AIDS [4,32]. The reduction in mortality is expected to improve in the future since many health policy decisions, care and management have been devolved to the county levels with a view of tailoring solutions to suit local needs [33,34].

ITN ownership was not related to SPR. The expected individual effect of ITNs on mortality could have been lost due to aggregating the data at village level. Distance to health facilities was an important factor in determining all-cause mortality across all age groups; showing that relatively small differences in distance can substantially affect mortality, especially in younger children. A study in the KHDSS on pediatric hospitalization argued that longer distances act as barriers to seeking care making people stay away even if they are sick [35]. Access to antenatal services, which is an important factor for neonatal survival is largely influenced by distance to health facilities [36] and may explain the large effect among neonates. Comparable to our rural setting, other studies have found similar results in sub-Saharan Africa [37–40].

People at lower socio-economic status experienced relatively higher mortality compared to those at a higher status. The effect was important among neonates for all-cause mortality and in children under the age of five for both all-cause and malaria specific mortality. This is consistent with other studies done in similar HDSS settings [41]. The poorest would most likely live in houses that are not well constructed hence offering little protection against vectors and are less likely to pay for effective treatment. This vulnerability is more pronounced in younger children even with small scale economic differences visible through simple asset ownership [42].

Altitude’s negative association with both all-cause and malaria-specific mortality supports findings showing that malaria vector abundance reduces with altitude [43]. Previous studies in the western Kenya region indicated that middle level and low altitude areas experienced higher mortality rates compared to the higher regions [40].

A strong spatial variation was estimated in the malaria specific model for infants, children u 1–4 years and overall model, suggesting that mortality is influenced by spatially structured exposures. This finding has also been noted in previous studies in the same region and other places in sub-Saharan Africa [5,6,40,44]. We also noted that for all age groups, spatial variation was higher than temporal variation signifying a reduced seasonal influence on mortality compared to spatial variability.

The use of verbal autopsy as a tool for determining cause of death has been criticized as not being very specific and can either under-estimate or over-estimate malaria [45]. The InterVA tool for verbal autopsy has however undergone rigorous tests and improvements recently to take care of physician failings, differences in high and low malaria transmission areas and found to be in high concordance with physician coding [46]. It also agrees with other population based projections in determining malaria as a cause of deaths, hence a useful tool for ascertaining cause of death in low income settings that lack proper vital registration systems. In our study, the SPR estimates may be biased due to missing patients who do not make it to health facilities, more so the older ages with reported lower health seeking behavior for fever related illnesses relative to younger children [47]. At the same time, microscopy case confirmation has been shown to have lower sensitivity [48] and the SPR may be influenced by changes in other febrile illnesses by inflating the denominator [9]. Using SPR from children below the age of 14 years as an indicator for transmission in the whole population could result in a bias since the behavioural and biological characteristics of children may not match exactly those of the whole population. However, we anticipated that the SPR of adults would be correlated at village level with that of children under the age of 14 as they come from the same community as the adults from whom we infer association between under 14 children’s SPR and adult mortality due to the strong environmental, climatic and other spatially explicit factors that drive malaria transmission. The sudden rise of SPR although not due to changes in the diagnostic methods could have been due to unobserved effects due to changes in health systems and may have biased the results to reflect a stronger effect in the association with mortality. By using longitudinal data over a long period of time, consistent methodology, rigorous quality control for microscopy diagnosis and assuming that SPR represents the level of malaria exposure in the population, we offset some of the shortcoming of using sentinel health facilities data.

## Conclusion

Our study showed that slide positivity rate is significantly associated with all-cause/malaria-specific mortality in this region of western Kenya. By quantifying excess mortality due to malaria, we show that small changes in malaria incidence can substantially reduce deaths.

As the fight towards malaria control and elimination continues, incidence risk data from sentinel health facilities can be used as a measure of exposure to assess, monitor and quantify both all-cause and malaria-specific mortality in low resource settings.

## Supporting information

S1 File Spatial and temporal description of data used in the study. (DOCX)

S2 File Bayesian model formulation. (DOCX)

S3 File Top cause of death by age groups. (XLSX)
