# Supplementary material for: Bayesian spatio-temporal modeling of mortality in relation to malaria incidence in Western Kenya
Source: PLoS One. 2017 Jul 13;12(7):e0180516. doi: 10.1371/journal.pone.0180516 (PMC5509217; doi:10.1371/journal.pone.0180516)
Supplement: S1 File — (DOCX) [file pone.0180516.s001.docx]

Spatial and temporal description of data used in the study

| **Data type** | **Description** | **Source** | **Temporal resolution** |
| --- | --- | --- | --- |
| Malaria slide positivity rates | Measured as the number of malaria positive slides out of the total blood slides examined by light microscopy. | Sentinel health facilities | Continuously (Jan 2007-Dec2012) |
| Cause of death (Malaria specific) | Deaths of registered KHDSS residents are reported continuously by village reporters based in the same village as soon as possible. A verbal autopsy is then done by a trained interviewer from the most appropriate interviewee who was closest to the deceased and knew about the illness, disease or condition that led to death. Using the InterVA model this data is used to determine the most probable causes of death from which malaria as a cause is determined. | Household level | Continuously (Jan 2007-Dec2012) |
| Socioeconomic status | Constructed based on household asset ownership using a composite score, the multiple correspondence analysis (MCA) technique and categorized into 3 quintiles as least poor for the well off, poor for the average and poorest for the lowest rank | Household level | Yearly (2007-2012) |
| Person time | The HDSS collects data on an initial population at the start of the observation period followed by subsequent 4 monthly surveillance cycles that provide data on births, deaths, in-migration and out-migrations. Using these, person time of observation in years was calculated as the total time spent by a registered KHDSS resident in the study area during the study period | Household level | Continuously (Jan 2007-Dec2012) |
| Bed net ownership | Calculated as the percentage of households per village owning at least one net for every two people | Household level | Yearly (2007-2012) |
| Altitude | Extracted from the Shuttle Radar Topography Mission (SRTM) by the U.S. Geological Survey - Earth Resources Observation and Science (USGS EROS) Data Center (https://eros.usgs.gov/elevation-products) | Household level | Once |
| Distance to health facility | Calculated as the Euclidean difference between the household and the nearest health facility in kilometers, aggregated at village level and classified as less than 1km, 1 to 2km and greater than 2km | Household level | Once |
| All-cause mortality | From the KHDSS continuous 4 monthly cycles, data on all deaths among the registered residents is collected to provide the number of deaths in the population and by age groups.. | Household level | Continuously (Jan 2007-Dec2012) |
