# Supplementary material for: Bayesian spatio-temporal modeling of mortality in relation to malaria incidence in Western Kenya
Source: PLoS One. 2017 Jul 13;12(7):e0180516. doi: 10.1371/journal.pone.0180516 (PMC5509217; doi:10.1371/journal.pone.0180516)
Supplement: S2 File — (DOCX) [file pone.0180516.s002.docx]

**S2 Bayesian model formulation.**

Let **Y***_jt_* be the average number of deaths (all-cause or malaria-specific) in village *j* at time interval *t*. We assume that **Y***_jt_* arises from a negative binomial distribution.

$\mathbf{Y}_{jt}\sim dnegbin(\mathbf{P}_{jt},\boldsymbol{r)}$

where P*_jt_*, is the proportion of deaths occurring in village *j* at time interval *t* and *r* is the dispersion parameter with,

$\mu_{j}=r\frac{1-p}{p}$ and$\sigma_{i}^{2}=r(1-p)p^{-2}$.

We modeled the association above between covariates (*X*) and mortality status of individuals by village on the logit, as

$logit\left( \mu_{jt} \right)=logit\left( N_{jt} \right)+\beta_{0}+{\sum_{1}^{k} \beta_{u}X}_{u}+\phi_{j}+\varepsilon_{t}$ , *u=1,2,……k*

where $\mu_{jt}$ is the number of deaths in each village at time *t*, $N_{jt}$the total person time contributed by persons in each village as discrete months, $\beta_{i}$ the regression coefficients, $\phi_{j}$ the village specific spatial effects and $\varepsilon_{t}$ the temporal (monthly) random effects.

We assumed that $\phi_{j}$ are parameters from a latent spatial process modelled by a Gaussian distribution with covariance matrix quantifying the relation between any pair of villages as a function of their distance irrespective of the direction using an exponential correlation function, that is $\emptyset\sim MVN(0,\sum)$, $\sum_{kl}= \sigma_{1}^{2} exp(-\rho d_{kl})$ where $\sigma_{1}^{2}$ is the spatial variation, $d_{kl}$ is the distance between villages *k* and *l*, and $\rho$ is the rate of correlation decay with increasing distance. The minimum distance at which the spatial variation is less than 5% is called range and can be obtained from the value $3/\rho$ (49). Temporal effect ($\varepsilon_{t}$) was modeled by an autoregressive process of order 2. We specified non-informative normal prior distributions with mean zero and large variance for the *β_i_* *i*=1,…… regression coefficients, an inverse gamma prior for $r \sim IG(1.01, 0.001)$, an inverse gamma priors for $\sigma_{e}^{2}$ and $\sigma^{2}$. A gamma prior for $\rho$, that is $\sigma_{e}^{2} {, \sigma}^{2} \sim IG(2.01, 1.01)$ and $\rho\sim G(0.01, 0.01)$.

The model was fitted using Markov Chain Monte Carlo (MCMC) simulation algorithm in OpenBugs version 3.1.2 (Imperial College and Medical Council, London, UK) to estimate model parameters (50). We ran a single chain sampler discarding the first 10,000 iterations. Convergence was assessed by Gelman-Rubin diagnostic (51) and attained at 100,000 iterations.
